# On the Paramagnetic-Like Susceptibility Peaks at Zero Magnetic Field in $\hbox{WSe}_{2-x}\hbox{Te}_{x}$ Single Crystals

**DOI:** 10.1186/s11671-022-03743-y

**Published:** 2022-11-10

**Authors:** Shiu-Ming Huang, Pin-Cing Wang, Kuo-Yi Hung, Fu-En Cheng, Chang-Yu Li, Mitch Chou

**Affiliations:** 1 Department of Physics, National Sun Yat-Sen University, 80424 Kaohsiung, Taiwan; 2 Department of Materials and Optoelectronic Science, National Sun Yat-Sen University, 80424 Kaohsiung, Taiwan; 3 Center of Crystal Research, National Sun Yat-Sen University, 80424 Kaohsiung, Taiwan

**Keywords:** Paramagnetic susceptibility peak, Topological material, Dirac point, Lattice distortion

## Abstract

A weakly temperature-dependent paramagnetic-like susceptibility peak at zero magnetic field is observed in $\hbox{WSe}_{2-x}\hbox{Te}_{x}$ with only marginal amount of ferromagnetic impurities. The ferromagnetic hysteresis loop and the magnetic moment splitting between zero-field-cooled and field-cooled processes indicate ferromagnetism in the samples. The paramagnetic-like susceptibility peak height is proportional to the remanent magnetic moment of hysteresis loops. High-resolution transmission electron microscope image supports that the observed ferromagnetic feature originates from lattice distortion. These results imply that the weakly temperature-dependent paramagnetic-like susceptibility peak originates from weak lattice distortion and/or superparamagnetism.

## Introduction

A paramagnetic susceptibility peak at zero magnetic fields is reported in various kinds of topological materials [1–6]. It is speculated to originate from the spin texture at the Dirac point of the topological surface state [7, 8]. According to this explanation, the spin couples to the momentum with a particular helicity in the topological surface state, making the spin align perpendicular to the momentum. However, at the Dirac point, spins are free to align along any direction. The freely aligned carrier spin will be polarized under external magnetic fields and that leads to the paramagnetic susceptibility peak. Following this model, a system with Fermi level laying above the topological surface Dirac point is a fundamental requirement for realizing this behavior. It is commonly known that the Fermi level is sensitive to the element components and sample fabrication conditions. However, none of the previous works provide direct evidence for the Fermi levels being above the Dirac point [1–6]. Without this evidence, the spin texture mechanism for the paramagnetic susceptibility peak is dubious. Furthermore, a recent report showed a paramagnetic susceptibility peak at zero magnetic field in a $\hbox{Bi}_{0.3}\hbox{Sb}_{1.7}\hbox{Te}_{3}$ topological insulator in which the Fermi level was 80 meV below the Dirac point at liquid nitrogen temperature [9]. This fundamentally violates the theoretical concept and completely breaks the spin texture model. This motivates us to investigate the relation between paramagnetic-like susceptibility and intrinsic ferromagnetism in topological materials.

A wide range of studies have reported that the Zigzag edge structure, lattice distortion and structure vacancies can lead to intrinsic ferromagnetism in topological materials and two-dimensional transition-metal dichalcogenides (2D TMDs) at room temperature [10–22]. To realize these ferromagnetic characteristics, several artificial treatments, such as atom replacement and lattice distortion, are performed.

In order to semi-qualitatively clarify the dependence of paramagnetic-like susceptibility and lattice-distortion-induced ferromagnetism, we demonstrate this through element replacement instead of the specific edge structures or vacancies. The magnetic characteristics of $\hbox{WSe}_{2}$, $\hbox{WSe}_{1.9}\hbox{Te}_{0.1}$ and $\hbox{WSe}_{1.8}\hbox{Te}_{0.2}$ single crystals were studied. The experimental results reveal ferromagnetic hysteresis loops, and the magnetic moment splits between zero-field-cooled and field-cooled processes. The paramagnetic-like susceptibility peak height is proportional to the remanent magnetic moment of hysteresis loops. The high-resolution transmission electron microscope image supports that the observed ferromagnetic feature originates from element replacement-induced structure distortion. The experimental result reveals that higher lattice disorder enhances ferromagnetic features. The weakly temperature-dependent paramagnetic-like susceptibility is observed in $\hbox{WSe}_{2}$, $\hbox{WSe}_{1.9}\hbox{Te}_{0.1}$ and $\hbox{WSe}_{1.8}\hbox{Te}_{0.2}$ single crystals. These results imply that instead of the spin texture model, the weakly temperature-dependent paramagnetic-like susceptibility peaks originate from weak lattice distortion.

## Experimental Methods

Chemical vapor transport (CVT) is adopted to grow tungsten diselenide doped with tellurium $\hbox{WSe}_{2-x}\hbox{Te}_{x}$ single crystal. 99.99% tungsten powder, selenium and tellurium were introduced into a silica ampoule. The sample space was then evacuated to a pressure of $10^{-3}$ torr. The first step is to synthesize the raw materials into polycrystalline powder. The ampoule was slowly heated to $600\,^{\circ}\hbox{C}$ over 95 h. Secondly, the sample was annealed at $1050\,^{\circ}\hbox{C}$ for 96 h. Finally, the annealed polycrystalline materials were sealed into a 20 cm silica tube. It was then placed in the two-zone furnace and raised the temperature to $1020\,^{\circ}\hbox{C}$ and gradually decrease the temperature to $980\,^{\circ}\hbox{C}$ in 170 h. After growth, the crystals were furnace cooled to room temperature. The as-grown crystals were cleaved along the basal plane, using a silvery reflective surface, and then prepared for further experiments. Energy-dispersive X-ray spectroscopy (EDS) exhibited that the crystal is $\hbox{WSe}_{2}$, $\hbox{WSe}_{2.2}\hbox{Te}_{0.1}$ and $\hbox{WSe}_{2.1}\hbox{Te}_{0.2}$.

The X-ray diffraction (XRD) was performed in the Bruker D2 Phaser X-ray diffractometer using the Cu K$\alpha$ radiation with a scan step of $0.01^{\circ }$. Raman spectroscopy was performed in the HORIBA, HR 800 with wavelength 633 nm and scan step of $0.3\,\hbox{cm}^{-1}$. Magnetic measurements were performed using the standard technique in a SQUID MPMS-3 magnetometer (Quantum Design). The highest magnetic field is 3 T, and the magnetic field step is 0.005 T.

## Results and Discussion

**Fig. 1:**
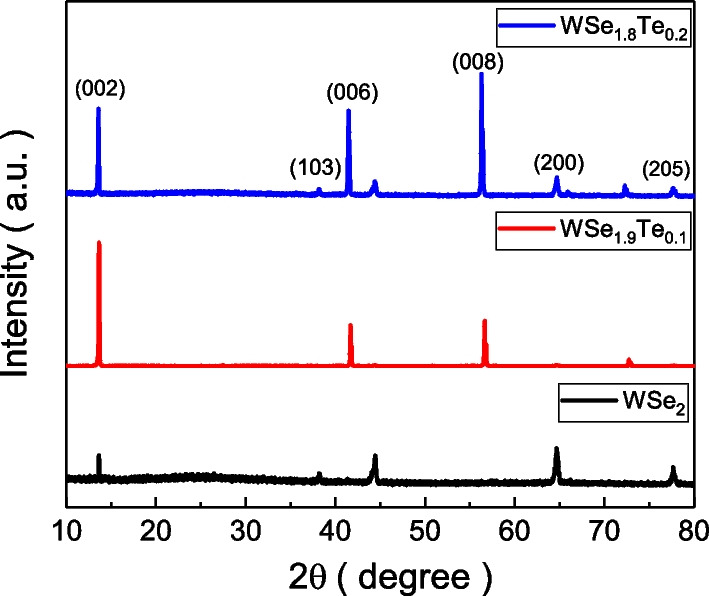
The XRD spectra of the $\hbox{WSe}_{2}$, $\hbox{WSe}_{1.9}\hbox{Te}_{0.1}$ and $\hbox{WSe}_{1.8}\hbox{Te}_{0.2}$ single crystals. The peak positions are consistent with the database of the $\hbox{WSe}_{2}$ crystal. The peak shift is observed in the $\hbox{WSe}_{1.9}\hbox{Te}_{0.1}$ and $\hbox{WSe}_{1.8}\hbox{Te}_{0.2}$. The shift of the diffraction peaks to lower angles in the crystal with more Te atoms replacement reflects an enlargement of the lattice constants with increasing Te content. The X-ray diffraction database is JCPDS No. 89-5257

Figure 1 shows the XRD spectra of $\hbox{WSe}_{2}$, $\hbox{WSe}_{1.9}\hbox{Te}_{0.1}$ and $\hbox{WSe}_{1.8}\hbox{Te}_{0.2}$ single crystals. The peak positions are labeled and they are consistent with the database of the $\hbox{WSe}_{2}$ crystal, indicating that it is a hexagonal structure [23]. The XRD peak shifts because of lattice distance change due to Te substitution. Te atom has a larger atom size than Se atom, and the lattice distance would be expected to be larger in the system with more Te atom substitution. This makes the XRD peak shift to lower $2\theta$. The peak (002) reveals shifts in these samples, and larger shifts are observed in the crystal with more Te atom replacement. The peak shift is roughly $0.06^{\circ }$ between $\hbox{WSe}_{2}$ and $\hbox{WSe}_{1.8}\hbox{Te}_{0.2}$.

**Fig. 2:**
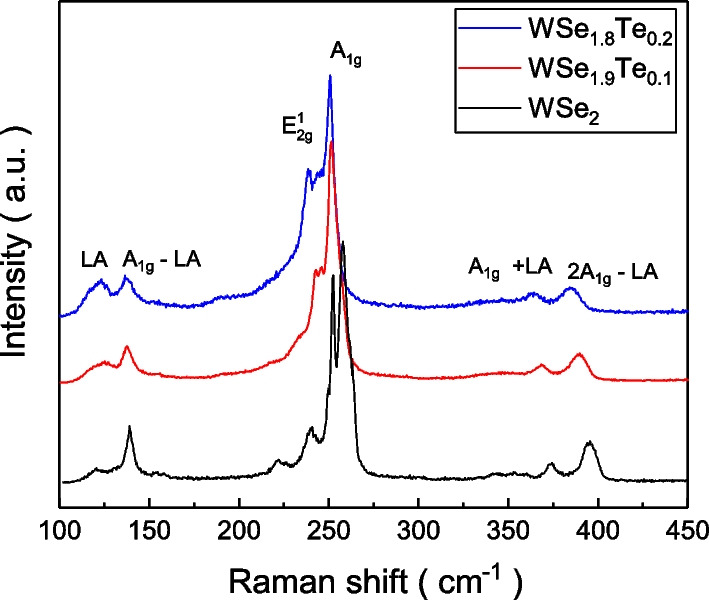
The Raman spectra of the $\hbox{WSe}_{2}$, $\hbox{WSe}_{1.9}\hbox{Te}_{0.1}$ and $\hbox{WSe}_{1.8}\hbox{Te}_{0.2}$ single crystals. Peaks shift to lower Raman shift in samples with more Te atom replacement

The Raman spectrum is sensitive to lattice vibration and is therefore an appropriate tool to detect the chemical bonding configuration. To identify the influence of the Te atom replacement on the lattice structure, the Raman spectra were obtained in the $\hbox{WSe}_{2}$, $\hbox{WSe}_{1.9}\hbox{Te}_{0.1}$ and $\hbox{WSe}_{1.8}\hbox{Te}_{0.2}$ samples. Figure 2 shows the Raman spectra. The $\hbox{WSe}_{2}$ exhibits three main peaks LA, E$^{1}_{2g}$ and A$_{1g}$ peaks at $120\,\hbox{cm}^{-1}$, $251\,\hbox{cm}^{-1}$ and $257\,\hbox{cm}^{-1}$, respectively [24]. In addition to the three main peaks, the spectrum exhibits three other peaks at $140\,\hbox{cm}^{-1}$, $373\,\hbox{cm}^{-1}$ and $395\,\hbox{cm}^{-1}$ and these peaks correspond to the $\hbox{A}_{1g}$-LA, $\hbox{A}_{1g}+\hbox{LA}$ and $2\hbox{A}_{1g}-\hbox{LA}$, respectively. A shift toward lesser value appears with increasing Te concentration. This implies that Te atoms replace Se atoms in the $\hbox{WSe}_{1.9}\hbox{Te}_{0.1}$ and $\hbox{WSe}_{1.8}\hbox{Te}_{0.2}$ crystals. The Raman peak height ratio of $\hbox{A}_{1g}/\hbox{E}^{1}_{2g}$ is mainly related to the atom vibration mode, and $\hbox{A}_{1g}/\hbox{E}^{1}_{2g}$ could be used as a factor to gauge the structure ordering.

**Fig. 3:**
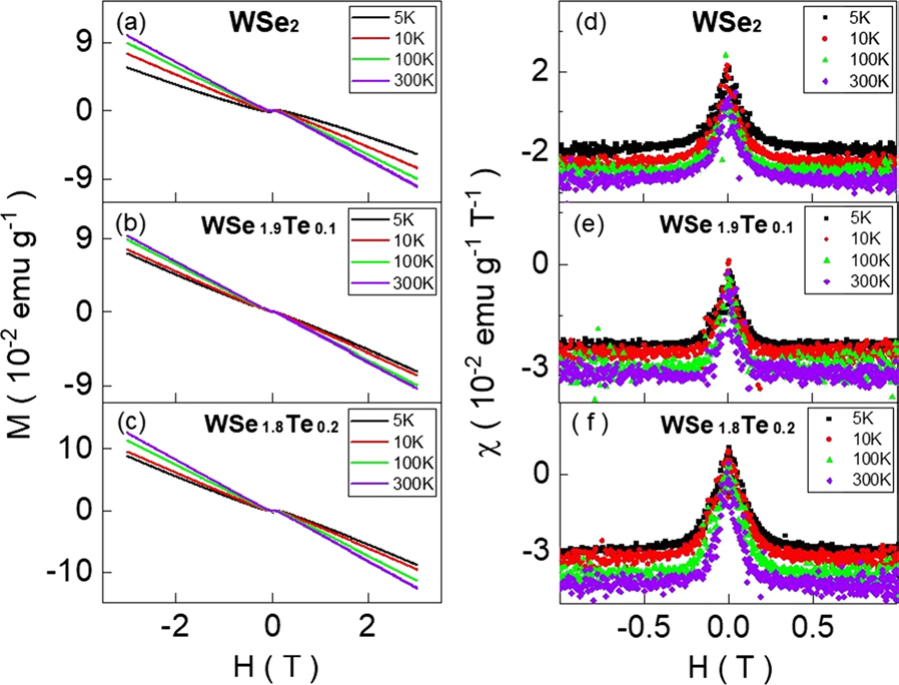
**a**–**c** show the magnetization as a function of magnetic field. They show paramagnetism at low magnetic field and diamagnetism at high magnetism at different temperatures. The diamagnetism is negatively proportional to the magnetic field. **d**–**f** show susceptibility peaks at a function of magnetic field. It reveals peaks at zero magnetic field, and the susceptibility peaks show weak temperature dependence from 5 to 300 K

Figure 3a–c exhibits the $M-H$ curves of $\hbox{WSe}_{2}$, $\hbox{WSe}_{1.9}\hbox{Te}_{0.1}$ and $\hbox{WSe}_{1.8}\hbox{Te}_{0.2}$. *M* is the measured magnetization, and *H* is the applied magnetic field. The magnetization shows a weak field dependence near zero magnetic field. It shows paramagnetism behavior at low magnetic field and diamagnetism at high magnetic field. The diamagnetism is negatively proportional to magnetic field at high magnetic field, and smaller susceptibility is observed at higher temperatures. Figure 3d–f shows the susceptibility as a function of magnetic field, and it reveals a paramagnetic-like peak at $H=0$. The peak values are of weak temperature dependence. This paramagnetic-like susceptibility peak at $H=0$ is widely speculated to originate from the spin texture at the Dirac point of surface state in topological materials [1–6]. The carrier spin couples to the carrier momentum and that leads to the spin–momentum locking [7, 8]. The surface state carrier possesses a particular spin helicity. It makes spins randomly distributed at the Dirac point of the topological surface state because of the different spin textures above and below the Dirac point. The randomly distributed spins will align up with external magnetic field and form a paramagnetic-like susceptibility peak at $H=0$. However, no one has provided this evidence in all previous reports [1–6]. This speculation is still under doubt due to the lack of direct evidence of the existence of the Dirac point. Following this theoretical model, one would not detect the paramagnetic-like peaks without the Dirac point. Moreover, a report revealed similar paramagnetic-like peaks in a $\hbox{Bi}_{0.3}\hbox{Sb}_{1.7}\hbox{Te}_{3}$ topological insulator in which the angle-resolved photoemission spectroscopy result revealed that the Fermi level was located below the Dirac point [9], making this explanation obsolete.

The paramagnetic-like susceptibility peak height at $H=0$ in the $\hbox{WSe}_{2}$, $\hbox{WSe}_{1.9}\hbox{Te}_{0.1}$ and $\hbox{WSe}_{1.8}\hbox{Te}_{0.2}$ is weakly temperature dependent, similar to the reported singular paramagnetic behavior in topological materials. The $\hbox{WSe}_{2}$ exhibits the topological characteristics in the octahedral 1T phase, and conventional semiconductors in the hexagonal 2H phase [25]. Since 1T phase only exists in monolayer single crystal, the phase structure of our $\hbox{WSe}_{2}$ single crystal bulk is 2H. Therefore, our $\hbox{WSe}_{2}$ single crystal should exhibit no topological characteristics. Following the postulated spin texture model, the paramagnetic-like susceptibility peak at $H=0$ should not be observed in our $\hbox{WSe}_{2}$. This observed paramagnetic-like peak in our $\hbox{WSe}_{2-x}\hbox{Te}_{x}$ bulk single crystal must originate from other mechanisms.

**Fig. 4:**
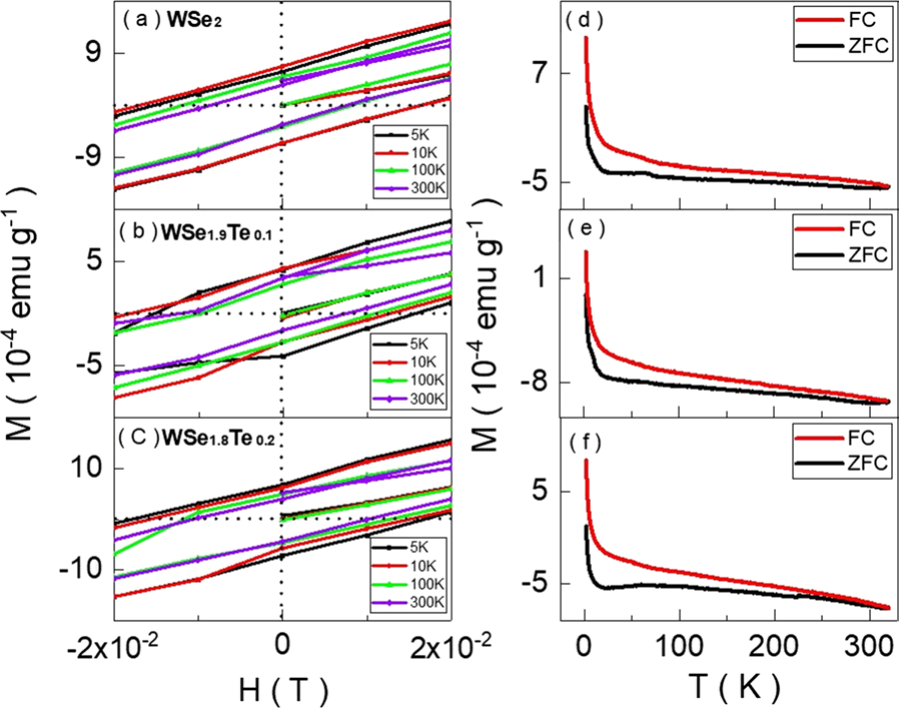
**a**–**c** show the magnetization as a function of magnetic field. It reveals magnetic hysteresis loops, and the coercive field is roughly 0.02 T. **d**–**f** show the temperature-dependent magnetization of field-cooled and zero-field-cooled processes. It splits from 330 to 2 K. **a**–**f** support the ferromagnetic characteristic in the $\hbox{WSe}_{2-x}\hbox{Te}_{x}$ single crystals

Recently, it was speculated that the paramagnetic-like susceptibility peak is a consequence of ferromagnetism [10]. Figure 4a–c exhibits the $M-H$ curves of $\hbox{WSe}_{2}$, $\hbox{WSe}_{1.9}\hbox{Te}_{0.1}$ and $\hbox{WSe}_{1.8}\hbox{Te}_{0.2}$ in a narrow scale ranges. It is expanded from Fig. 3a–c. They show hysteresis loops in $\hbox{WSe}_{2}$, $\hbox{WSe}_{1.9}\hbox{Te}_{0.1}$ and $\hbox{WSe}_{1.8}\hbox{Te}_{0.2}$ crystals. The coercive field is roughly $\pm 0.02\,\hbox{T},$ and it is insensitive to temperature. To further confirm the existence of ferromagnetism, the field-cooled (FC) and zero-field-cooled (ZFC) processes are performed. Figure 4d–f shows the magnetization of FC and ZFC processes is split and that supports the ferromagnetic feature in the $\hbox{WSe}_{2-x}\hbox{Te}_{x}$ single crystals from 2 to 330 K. These results imply that in contrast to the proposed spin texture model, the paramagnetic-like peak is a ferromagnetic feature.

The magnetic impurities can lead to ferromagnetism in ordinary materials. The X-ray photoelectron spectroscopy (XPS), electron probe microanalyzer (EMPA) and inductively coupled plasma mass spectrometry (ICPMS) analysis are performed to identify the source of the observed ferromagnetism, and the results exhibit no detectable ferromagnetic impurities. The ICPMS shows the magnetic element concentration is lower than 0.2 ppm in each sample.

**Fig. 5:**
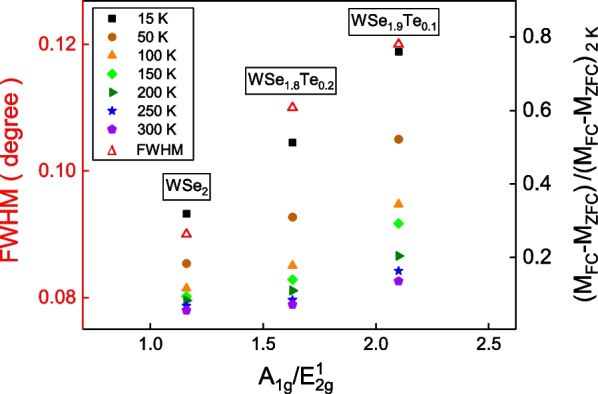
The full width at half maximum (FWHM) of the X-ray diffraction peak (002), and the normalized magnetic moment difference of field-cooled (FC) and zero-field-cooled (ZFC) processes as a function of $\hbox{A}_{1\textrm{g}}/\hbox{E}^{1}_{2\textrm{g}}$. Both follow the same tendency and are positive in the $\hbox{A}_{1\textrm{g}}/\hbox{E}^{1}_{2\textrm{g}}$. Larger lattice dislocation leads to larger FWHM. These results imply the element replacement distorts the lattice and enhances intrinsic ferromagnetism

Lattice dislocation directly affects the lattice vibration mode. The Raman peak height ratio of $\hbox{A}_{1\textrm{g}}/\hbox{E}^{1}_{2\textrm{g}}$ is a factor to gauge the lattice distortion. To semiquantitatively confirm that the intrinsic ferromagnetism originates from the element replacement-induced lattice distortion, the full width at half maximum (FWHM) of the X-ray peak (002) and the normalized magnetic moment difference of FC and ZFC processes are plotted as a function of $\hbox{A}_{1\textrm{g}}/\hbox{E}^{1}_{2\textrm{g}}$. Figure 5 shows that the full width at half maximum (FWHM) of the X-ray peak (002) and the normalized magnetic moment difference of FC and ZFC follow the same tendency, and both exhibit a positive dependence on the $\hbox{A}_{1\textrm{g}}/\hbox{E}^{1}_{2\textrm{g}}$. Larger FWHM implies larger lattice dislocation or crystal distortion. These results imply that the Te atoms replacement and lattice distortion enhance intrinsic ferromagnetism which is insensitive to temperature. The element replacement-induced ferromagnetism has been experimentally performed in several systems. The ferromagnetism was studied in $\hbox{MoS}_{2-x}\hbox{Se}_{x}$ crystals, and it reveals that the ferromagnetism is sensitive to the $\textrm{Se}/\textrm{S}$ ratio. It exhibits the largest ferromagnetism in the $\hbox{Mo}(\hbox{S}_{0.49}\hbox{Se}_{0.51})_{2}$ nanosheet [26]. This supports that similar to the element vacancy or the zigzag edge structure, element replacement and dislocation would enhance ferromagnetism [10–22]. Also, ferromagnetism and magnetoresistance hysteresis has been observed in molecular-beam epitaxy grown non-magnetic group IV $\hbox{Ge}_{1-x}\hbox{Sn}_{x}$ thin film. It forms a $\hbox{Ge}_{1-x}\hbox{Sn}_{x}$ alloy at the interface between Ge and Sn thin films. The observed ferromagnetism is understood as the inversion symmetry breaking from atomic disordering in the alloy [27]. These results support that the lattice distortion enhances ferromagnetism. Superparamagnetism is a form of magnetism which appears in systems with small ferromagnetic nanoparticles. In sufficiently small nanoparticles, magnetic moments can randomly flip direction under the influence of temperature. In the absence of an external magnetic field, their magnetic moments would be averaged to zero and an external magnetic field would be able to align the magnetic moments of the nanoparticles, similarly to paramagnetism. We note that system with ferromagnetic impurities whose concentration is lower than 0.2 ppm can still form superparamagnetism. The magnitude of M around $10^{-2}\,\hbox{emu}/\hbox{g}$ could be induced by the superparamagnetism arising from the nanoclusters of ferromagnetic elements. We infer from the previous studies and the experimental results shown above that the observed magnetic properties in our $\hbox{WSe}_{2-x}\hbox{Te}_{x}$ single crystals are ferromagnetism originated from the structural distortion due to Te replacement and/or superparamagnetism.

**Fig. 6:**
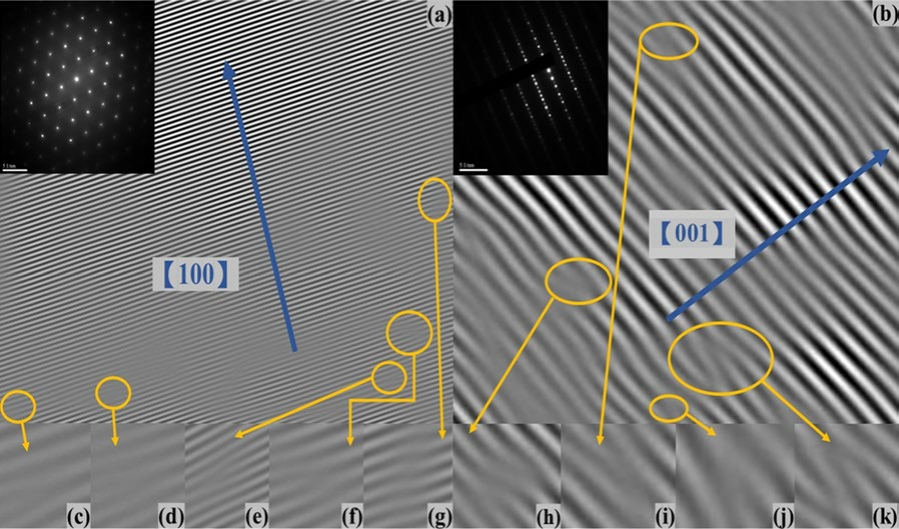
**a** and **b** The high-resolution transmission electron microscope image of the $\hbox{WSe}_{2}$ single crystals in different axes and planes. The insets are the reciprocal lattice image of selected area electron diffraction patterns of the $\hbox{WSe}_{2}$ single crystals in different axes and planes. **c**–**k** reveal lattice dislocation of the $\hbox{WSe}_{2}$ single crystals at several locations in different axes and planes

Figure 6a and b shows the high-resolution transmission electron microscope images of the $\hbox{WSe}_{2}$ single crystals in different axis and planes. The insets show the reciprocal lattice image of selected area electron diffraction patterns of the $\hbox{WSe}_{2}$ single crystals in different axes and planes. Figure 6c–k reveals lattice dislocation of the $\hbox{WSe}_{2}$ single crystals at several locations in different axes and planes. This agrees with the observed ferromagnetic behaviors in the $\hbox{WSe}_{2-x}\hbox{Te}_{x}$ single crystals being originated from the lattice dislocation. It is challenging to evaluate the ordered magnetic moment since the distribution of dislocations along with their individual contributions is not known. Instead of evaluating the magnetic moment of lattice dislocation, the remanent magnetization of hysteresis loop is an appropriate parameter to quantify the effective long-range ferromagnetic moment.

**Fig. 7:**
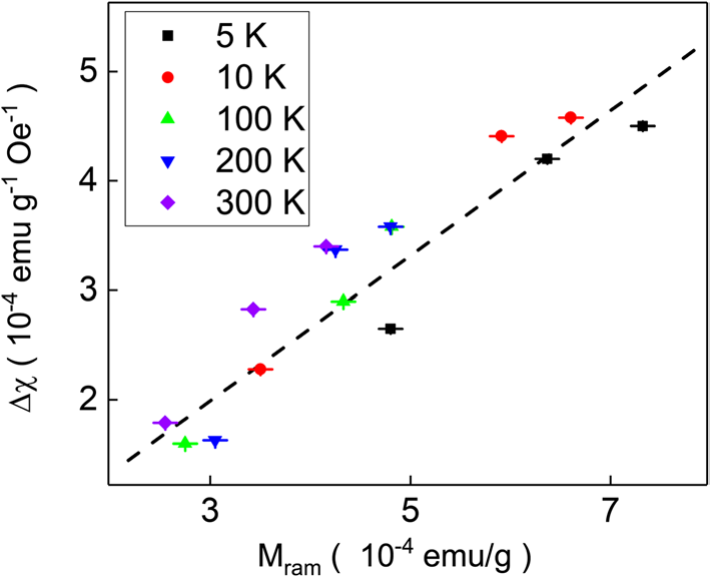
The paramagnetic-like susceptibility peak height as a function of remanent magnetization of hysteresis loops in the $\hbox{WSe}_{2-x}\hbox{Te}_{x}$ single crystals at different temperatures. The susceptibility peak height is proportional to the remanent magnetization of hysteresis loops. This supports that the observed weakly temperature-dependent paramagnetic-like susceptibility peak originates from intrinsic ferromagnetism

Figure 7 shows the paramagnetic-like susceptibility peak height as a function of remanent magnetization of hysteresis loops in the $\hbox{WSe}_{2-x}\hbox{Te}_{x}$ single crystals at different temperatures. It reveals that the paramagnetic-like susceptibility peak height is proportional to the remanent magnetization of hysteresis loops. This also implies that the lattice dislocation enhances ferromagnetism. This also implies that the susceptibility peaks at zero magnetic fields originate from the intrinsic magnetization induced by the crystal dislocation in different crystal axes and planes. The lattice distortions enhance the observed intrinsic ferromagnetism.

## Conclusion

We studied the magnetic characteristics of $\hbox{WSe}_{2}$, $\hbox{WSe}_{1.9}\hbox{Te}_{0.1}$ and $\hbox{WSe}_{1.8}\hbox{Te}_{0.2}$ single crystals with only marginal amount of ferromagnetic impurities. In addition to weakly temperature-dependent paramagnetic-like susceptibility peaks at zero magnetic fields, it reveals ferromagnetic hysteresis loops and magnetization split between zero-field-cooled and field-cooled processes. The hysteresis loop coercive fields and paramagnetic-like susceptibility peak height are both weakly temperature dependent. The X-ray diffraction peaks shift and Raman spectra peaks shift to lower Raman shift support the Te replacement in the $\hbox{WSe}_{2-x}\hbox{Te}_{x}$ single crystals. A high-resolution transmission electron microscope shows the weak lattice dislocation in different axes and planes. The full width of half maximum of X-ray diffraction and normalized magnetization split of zero-field-cooled and field-cooled follow the same tendency of the lattice dislocation degree which is defined as Raman spectrum peak height ratio, $\hbox{A}_{1\textrm{g}}/\hbox{E}^{1}_{2\textrm{g}}$. The weakly temperature-dependent paramagnetic-like susceptibility peak height is proportional to the remanent magnetization of hysteresis loops at temperatures in the $\hbox{WSe}_{2-x}\hbox{Te}_{x}$ single crystals. These results support that the observed weakly temperature-dependent paramagnetic-like susceptibility peaks originate from the intrinsic ferromagnetism induced by the weak lattice distortion and/or superparamagnetism.

## Data Availability

The datasets generated during and/or analyzed during the current study are available from the corresponding authors on reasonable request.

## Abbreviations

EDS: Energy-dispersive X-ray spectroscopy
XPS: X-ray photoelectron spectroscopy
EPMA: Electron probe microanalyzer
ICPMS: Inductively coupled plasma mass spectrometry
FWHM: Full width at half maximum.
